# Predictors of the Progression of Dementia Severity in Brazilian Patients with Alzheimer's Disease and Vascular Dementia

**DOI:** 10.4061/2010/673581

**Published:** 2010-03-14

**Authors:** Márcia L. Chaves, Ana L. Camozzato, Cristiano Köhler, Jeffrey Kaye

**Affiliations:** ^1^Dementia Outpatient Clinic and Behavioral Sciences Program, Neurology Service and Medical Sciences Post-Graduation Course, Hospital de Clínicas de Porto Alegre, Universidade Federal do Rio Grande do Sul School of Medicine, Porto Alegre 90035-903, Brazil; ^2^Dementia Outpatient Clinic and Behavioral Sciences Program, Neurology Service, Hospital de Clínicas de Porto Alegre, Universidade Federal do Rio Grande do Sul School of Medicine, Porto Alegre 90035-903, Brazil; ^3^Layton Aging and Alzheimer's Disease Center, Oregon Health & Science University, Portland, OR 97239, USA

## Abstract

*Introduction*. This study evaluates the progression of dementia and identifies prognostic risk factors for dementia. *Methods*. A group of 80 Brazilian community residents with dementia (34 with Alzheimer's disease and 46 with vascular dementia) was assessed over the course of 2 years. Data were analyzed with Cox regression survival analysis. *Results*. The data showed that education predicted cognitive decline (HR = 1.2; *P* < .05) when analyzed without controlling for vascular risk factors. After the inclusion of vascular risk factors, education (HR = 1.32; *P* < .05) and hypertension were predictive for cognitive decline (HR = 38; *P* < .05), and Alzheimer's disease diagnosis was borderline predictive (*P* = .055). *Conclusion.* Vascular risk factors interacted with the diagnosis of vascular dementia. Education was a strong predictor of decline.

## 1. Introduction

It is estimated that 24.3 million people suffer from dementia today, with 4.6 million new cases every year. Of those with dementia, 60% live in developing countries, and the rate of increase in prevalence is predicted to be three to four times higher in developing countries than in developed countries [1].

In most prevalence studies, Alzheimer's disease (AD) has been associated with 70% or more of all cases of dementia and the main contributor to the steep increase with age in the prevalence of dementia [2]. In turn, dementia with a vascular component comprises nearly half of all cases of dementia in persons aged 85 years and older [3]. Patients with vascular dementia (VaD) have poorer survival than those with AD: the median survival from dementia onset to death is 3.9 years for patients with vascular dementia (VaD) and 7.1 years for patients with AD [4]. 

It is important to evaluate the progression of dementia and to estimate predictors of cognitive and functional decline in patients with an established diagnosis of dementia. Gender, education, and time from AD onset did not significantly predict cognitive and functional decline in one study [5]. In another study, there were also no predictors of disease progression except for gender: men exhibited a greater rate of cognitive decline than women [6]. Another report demonstrated that, during the early and very late stages of AD, cognitive deterioration was slower than during the middle stages and no clinical variables other than the degree of cognitive impairment and previous rate of cognitive decline predicted deterioration [7]. 

Higher education and participation in prediagnostic activities have been associated with faster cognitive decline in many AD longitudinal studies [8–10]. In addition, psychotic symptoms and disruptive behavior predicted faster disease progression in both the cognitive and functional dimensions of AD [11]. Diffuse cognitive impairments predicted cognitive decline, and visuospatial deficits predicted functional progression in AD [12].

Among patients with VaD, longitudinal changes in instrumental and basic activities of daily living were most strongly associated with changes in executive functioning and memory abilities, respectively [13]. Independent of both level and rate of change of prestroke cognitive performance and other risk factors for cognitive decline, incident stroke was associated with more than twice the risk of subsequent dementia (hazard ratio, 2.1; 95% CI, 1.55 to 2.81). Among those persons who developed dementia after stroke, 58.2% were diagnosed with VaD, and 32.7% were diagnosed with AD [14]. High cerebrovascular disease burden in VaD was associated with the worst survival rate [15]. 

VaD and AD have been linked to a number of cardiovascular factors [16–20], but the effect of vascular risk factors on the progression of already-established dementia has been less studied. Vascular risk factors measured clinically and biochemically did not significantly increase the rate of deterioration during 18 months in AD patients with a low burden of cerebrovascular risk factors; however, cerebrovascular events predicted cognitive decline in AD [21]. Decreased cardiovascular reactivity, atrial fibrillation, systolic hypertension, and angina also predicted faster decline in AD patients [22, 23]. History of coronary artery bypass graft surgery, diabetes, and treatment with antihypertensive medications were associated with slower rate of decline [23]. On the other hand, a longitudinal study did not find an association between baseline cardiovascular risk factors and progression from CDR 1.0 to CDR 2.0 over an 18-month follow-up [24]. 

We hypothesized that VaD patients would present faster decline than AD patients. We also hypothesized that younger and more educated patients and those with higher exposure to vascular risk factors would present faster decline. As VaD patients presented with more vascular risk factors, we supposed that this combination would also increase the rate of progression of VaD.

The main objective of the present study was the evaluation of the progression of dementia (the severity of decline) in AD and VaD. Most patients were mildly to moderately demented at baseline. We also analyzed predictors of progression of severity of dementia.

## 2. Methods

Eighty AD and VaD patients were selected consecutively from the Dementia Outpatient Clinic and enrolled in a cohort study to evaluate the progression of dementia severity and clinical predictors of decline. This sample size was sufficient to detect 15% differences in risk for decline (*P* < .05). The diagnosis of AD followed the criteria of the NINCDS-ADRDA for probable AD. VaD was diagnosed by the National Institute of Neurological Disorders and Stroke-Association Internationale pour la Recherche et l'Enseignement en Neurosciences (NINDS-AIREN) criteria [25]. The Hachinski Ischemic Score (HIS) [26] was also applied in all patients to verify that an ischemic process was associated with VaD and to exclude ischemia in AD. 

The assessments were carried out every 6 months over 2 years. At the interviews, patients, corroborated by their caregivers, were evaluated for cognition and behavior. The following assessments were made at baseline. Demographic data, including educational attainment and Portuguese fluency, were collected for all patients. The risk factors for cerebrovascular disease, hypertension, and diabetes were evaluated by clinical history and physical examination. Hypertension was defined as systolic blood pressure of ≥140 mmHg, diastolic blood pressure of ≥90 mmHg, or by the use of prescribed antihypertensive medication. Diabetes mellitus was considered present if the patient had a fasting glucose level of ≥120 mg/dL or used antidiabetic medication. Family history of dementia and neuroimaging findings were also collected at study entry, and a neurological examination was performed. A senior neuroradiologist, working with dementia neuroimage for more than 5 years, blinded to the clinical data, performed the readings of CT scans. The analyses were qualitative. Atrophy was defined subjectively, based on telencephalic cortical sulci prominence and lateral ventricles diameter. Leukoaraiosis was defined as bilateral and nearly symmetrical areas of decreased density of the periventricular white matter and centrum semiovale. Focal abnormality was described as areas of hypodensity without leukoaraiosis, expressing ischemic lacunae, on neuroimage.

Baseline severity of dementia was assessed with the Clinical Dementia Rating (CDR) scale, applied by trained interviewers [27, 28]. The CDR was considered a categorical variable by coding mild, moderate and severe as 1, 2, and 3, respectively.

The detailed examinations at each follow-up assessment included medical, neurological, neuropsychiatric, and cognitive evaluations, by three neurologists and one psychiatrist. The CDR scale was also used at each follow-up assessment. 

The main outcome assessed was the disease progression in terms of dementia severity. The change from a milder category on CDR to a severer condition on this scale (i.e., a one-point drop in CDR) was considered an indicator of the progression of dementia severity. The study was approved by the Ethics Committee for Medical Research at the University Hospital where it took place. Informed consent was obtained from the subjects or their nearest relatives or both.

## 3. Statistical Analyses

Progression of dementia severity was evaluated by Cox regression survival analysis, and survival curves were derived with the Kaplan-Meier's method. The date of the follow-up when a one-point drop in CDR was recorded marked the occurrence of disease progression. The following variables were considered as predictors of disease progression: age, sex, education, diagnosis (AD or VaD), diabetes, hypertension, and neuroimaging findings at study entry. Two Cox proportional hazards models were performed to determine whether baseline variables could predict the progression of dementia. In the first model, age, sex, educational level, family history of dementia, neuroimaging findings, and diagnosis (AD or VaD) were considered as explanatory variables. In the second analysis, vascular risk factors were added as an explanatory variable to the previously used factors in the model.

All procedures were carried out with the Statistical Package for the Social Sciences (SPSS) 14 for Windows.

## 4. Results

The sample of the first evaluation (at entry to the study) was composed of 80 patients: 39 (49%) males and 41 (51%) females. The mean ± SD age at entry to the study was 69.9  ± 8.9 years (range: 50 to 87 years), and education level varied from 0 to 16 years of schooling (4.57 ± 4.02, mean ± SD). Of the 80 patients in our first analysis, 34 (43%) fulfilled criteria for probable AD, and 46 (57%) for probable VaD. All patients with VaD had a history of the previous stroke at baseline. Of the 80 patients enrolled in the study, 28 did not present any vascular risk factor. Hypertension was observed in 37 patients, diabetes in 6, and the association of hypertension and diabetes in 9. AD patients at baseline were mild (CDR = 1) (*n* = 20), moderate (CDR = 2) (*n* = 9), and severe (CDR = 3) (*n* = 5), and VaD patients were mild (CDR = 1) (*n* = 27) and moderate (CDR = 2) (*n* = 19). The sample included only community-dwelling patients (Table 1).

Progression of dementia severity during the 2-year follow-up was observed in 17 patients. No improvements were observed during the follow-up. Stroke occurrence in AD patients or reoccurrence in VaD patients was not observed during the 2-year follow-up.

In the first multivariate Cox model without vascular risk factors, education was the only significant variable (HR = 1.2; CI 95% 1.02–1.37; *P* = .024). One more year of education increased the risk (hazard ratio) 1-2-fold (Table 2).

However, in the second multivariate Cox model with the inclusion of vascular risk factors (hypertension, diabetes, and hypertension + diabetes), education and hypertension were significant factors. Diagnosis (AD or VaD) was not significant in the first model (*P* = .94), but showed a borderline effect in the second model (*P* = .055). For the second model, one year more of education increased the risk of dementia progression 1.32 times, and the presence of hypertension increased the risk 38 times (Table 3).

**Figure 1:**
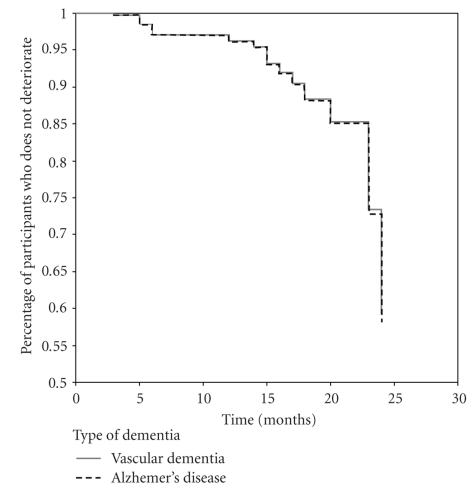
Survival Rate in Relation to Severity of Dementia (CDR scale): without vascular risk factor in the model.

**Figure 2:**
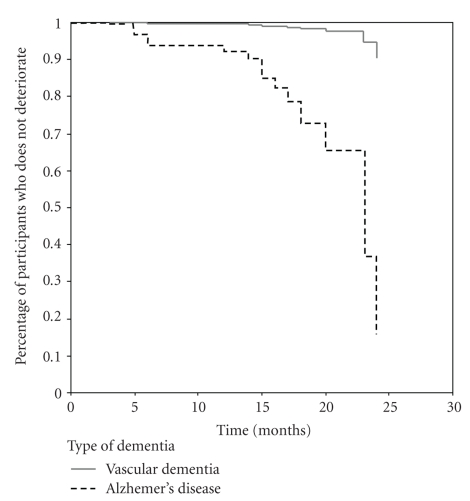
Survival Rate in Relation to Severity of Dementia (CDR scale): with vascular risk factor in the model.

Figures 1 and 2 display the rate of progression of AD and VaD obtained from the two Cox regression models. In Figure 1, the progression was similar between AD and VaD. On the other hand, Figure 2 demonstrates slow progression of VaD—almost 4/5 of the period of observation with acceleration at the end of the period—and fast progression of AD.

## 5. Discussion

This longitudinal study was designed to evaluate the rate of progression and predictors of progression of dementia severity. The main findings of the present study were the similar evolution of severity in both AD and VaD, and the impact of hypertension on the progression of dementia. When the course of VaD was analyzed without control for the vascular risk factors, the progression was similar to that of AD. After the addition of these factors—specifically hypertension—to the model, VaD presented slower progression (*P* = .055). Additionally, higher education was associated with faster disease progression in dementia patients in both mathematical models.

Educational level was an independent predictor of progression in both AD and VaD: the higher the education, the worse the severity of dementia. Other longitudinal studies have shown that highly educated patients and those who have had greater participation in prediagnostic intellectual activities have faster cognitive decline than less educated patients; however, these groups have similar mortality rates [9, 10]. Surrogates of higher cognitive reserve, such as higher educational level, higher occupational attainment, and higher premorbid reading activity, have been associated with faster cognitive decline in patients with dementia [29, 30]. Those individuals with greater cognitive reserve may tolerate a greater burden of neuropathologic conditions in AD before clinical disease expression; relatively advanced neuropathologic disease by the time of diagnosis may result in faster disease progression [9, 29–31]. Cognitive reserve may also have played a role in bearing the burden of a cerebrovascular mechanism disrupting cognitive processing until the point at which the patient manifests cognitive impairment. Higher education was associated with faster decline even in a sample presenting a relatively low level of education. In this case, a small difference in educational level is sufficient to contribute to the progression of dementia. Because developing and developed countries present distinct educational ranges, one could argue that this difference could reflect a generally faster severity of decline in developed countries.

Progression of disease severity in AD and VaD was similar during the period of observation without control for vascular risk factors. At the 12-month follow-up, about 5% of the participants had deteriorated; at 15 months, about 9%; at 20 months, 18%; at 23 months, 28%; at 24 months, 42% of the patients had already deteriorated. On the other hand, when controlled for vascular risk factors, the data showed that at 13 months of observation 10% of AD patients had deteriorated; at 15 months, 17%; at 12 months, 32%; at 23 months 37%; at 24 months, 84% of these patients had declined. Among the VaD patients, only 2% of the patients declined before month 23, and at 24 months, about 10% had already deteriorated. The slower progression of VaD was observed with statistical control for hypertension and educational attainment. Despite the borderline significance of the AD and VaD difference, this result deserves attention. Contrary to our previous hypothesis, we found a slower decline for VaD patients. We can hypothesize that hypertension, a stronger risk factor for the progression of dementia [19, 20, 32–34], masked the association of diagnosis (AD or VaD) with the progression of dementia. The adjusted model for hypertension revealed the faster decline of the AD patients. This finding is not surprising because hypertension is the main risk factor for stroke, and some types of stroke are more common causes of VaD. Therefore, the effect of hypertension is more important for the progression of VaD than for AD, and the mathematical control with the Cox regression analysis showed this difference. Hypertension is the major risk factor for VaD [34] and can be considered a prognostic factor for the progression of this disease.

Studies have previously evaluated prognostic factors for AD [6–11, 21–24] and for vascular dementia [13–16]. However, studies comparing the risk or prognostic factors in AD and VaD are scarce.

Hypertension is a direct risk factor for VaD [16, 18, 19, 34, 35], and other studies have suggested that hypertension also impacts the prevalence of AD [20, 32]; however, the effect of vascular risk factors on disease progression after established dementia remains to be elucidated. Our study highlights the significance of vascular risk factors, especially hypertension, on the progression of the severity of dementia, and the consequence of this finding is important. High blood pressure in midlife is associated with a higher incidence of both AD and VaD in later life in longitudinal studies [20, 32, 36, 37]. Chronically high blood pressure is associated with vessel wall thickening and narrowing of the vessel lumens in microvessels and large cerebral arteries. Rupture of the atheromatous plaques of larger cerebral arteries can cause complete occlusion of these arteries and infarction of the adjacent cerebral areas [38]. The ischemia and infarction of areas of the brain involved in memory and function increase the risk of vascular dementia [35]. Furthermore, hypoxia-induced factors derived from ischemia may potentiate amyloidogenic mechanisms and the development of AD [39]. We hypothesize that hypertension affects the progression of dementia by the same mechanisms involved in the pathogenesis and clinical manifestation of disease. High blood pressure is a main risk factor for white-matter lesions, which could promote faster progression of both VaD and AD. The atherosclerotic burden in the brain related to hypertension could also promote more rapid decline. Additionally, atherosclerosis and inadequate treatment of hypertension may induce cerebral hypoperfusion, ischemia, and hypoxia that could hasten cognitive decline. The treatment of the modifiable risk factors, such as hypertension, diabetes mellitus, hypercholesterolemia, and heart disease, is a strategy for the reduction of the risk of dementia [40–42]. Although we did not evaluate this issue, we may expect that the treatment of these vascular risks factors has a similar effect on slowing the progression of cognitive decline in dementia.

The strengths of the present study were the well-defined and well-evaluated vascular risk factors at entry to the study, the statistical model of prediction, the AD and VaD grouping sample, and the relevance of the main findings for developing countries. The definition and evaluation of vascular risk factors were designed to be comprehensive and reliable. We did not include the previous history or incident stroke as vascular risk factor because only VaD patients had previous history of stroke at baseline and incident stroke was not observed during the follow-up. The statistical model of prediction, adjustment, and control for variables, as well as the graphic illustrations, demonstrated very well what could have occur if the vascular risk factors, mainly hypertension, were actually controlled.

One limitation of the present study was that there was no distinction of VaD subtypes. Concept and definition of VaD are heterogeneous and the term VaD encompasses many different subtypes. These differences certainly have an impact on the clinical course of the disease. The various forms of VaD present different clinical, neuroimaging, and pathological features which may affect the clinical course in diverse ways. We used the NINDS/AIREN criteria to define vascular dementia, which is the most frequently used and the most restrictive. We also used the Hachinski Ischemic Score (HIS) to verify that an ischemic process was associated with VaD and to exclude ischemia in AD. Then, our results cannot be generalized to all definitions of VaD.

## 6. Conclusion

 This longitudinal study showed a similar severity of decline in both AD and VaD during two years. The impact of hypertension and education on the progression of dementia was also demonstrated. Vascular risk factors interacted with the diagnosis of VaD. Education was a strong predictor of decline. Finally, the importance of the present findings is reinforced by the information that disability has a greater absolute (and relative) impact on healthy-life expectancy at birth in poorer countries [43], and prevalence and rates of increase of dementia are higher in developing than in developed regions [1, 44]. Health public strategies to prevent and control treatable conditions such as hypertension may have an important impact on the burden of dementia in developing countries.

##  Competing Interests

The author(s) declare that they have no competing interests.

##  Author's Contributions

M. L. Chaves designed the study, was responsible for the statistical design of the study, supervised the data collection, and wrote the paper. A. L. Camozzato supervised the data collection, was responsible for carrying out the statistical analysis, and wrote the paper; C. Köhler collected the data and assisted with statistical analysis; J. Kaye assisted with writing the paper.

## Figures and Tables

**Table 1:** Demographic and clinical data at study entry.

Variable	Alzheimer (*N* = 34)	Vascular (*N* = 46)
Age (in years) mean ± SD (range)	73.23 ± 7.72 (60–87)	67.41 ± 9.08 (50–86)

Sex		
Male	11 (32%)	28 (61%)
Female	23 (68%)	18 (39%)

Educational Attainment (in years) mean ± SD (range)	4.30 ± 3.99 (0–16)	4.76 ± 4.06 (0–16)

Vascular risk factors		
None	28 (82%)	—
Hypertension	04 (12%)	33 (72%)
DM	02 (6%)	04 (9%)
Hypertension + DM	00 (0%)	09 (20%)

Family history		
No	18 (53%)	24 (52%)
Yes	11 (32%)	08 (17%)
Uncertain	05 (15%)	14 (30%)

Duration of follow-up (in months) mean ± SD (range)	11.01 ± 6.19 (3–25)	11 ± 6.31 (3–27)

Neurological Examination		
Normal	34 (100%)	
Neurological sign (hemiparesis, lower facial		
weakness, Babinski sign, sensory deficit,	—	25 (59%)
hemianopia, and dysarthria)*		
Other discrete symptoms-		19 (41%)
(mild asymmetric deep tendon reflex, tremor)		

Hachinski ischemic score mean ± SD (range)	2.90 ± 0.6 (1–4)	9.13 ± 2.61 (5–19)

CT scans:		
Normal	19 (56%)	—
Mild diffuse atrophy AND/OR ↑ ventricles	15 (44%)	—
Atrophy + leukoaraiosis	—	12 (26%)
Focal abnormality	—	34 (74%)

DM: Diabetes Mellitus; *NINDS-AIREN suggested symptoms.

**Table 2:** Predictive factors in Cox Proportionate Hazards Models, including diagnosis of dementia, age, educational level, sex, family history of dementia, and CT scan findings, for the progression of dementia (worsening of severity according to CDR scale).

Variable	Hazards ratio	CI 95% lower–upper	*P*-value	*r*
Diagnosis of dementia	0.949	0.30–3.05	.939	−0.053
Age	1.049	0.96–1.15	.318	0.048
Sex	0.433	11–1.70	.230	−0.838
Educational level	1.185	1.02–1.37	.024	0.170
Family history of dementia			.785	
Present	0.983	0.24–4.08	.982	−0.017
Unknown	0.559	0.11–2.89	.487	−0.581
CT findings			.881	
Mild diffuse atrophy AND/OR ↑ ventricles	15831.99	0.00–6.2*E* ^+096^	.929	9.670
Atrophy + leukoaraiosis	34834.69	0.00–1.4*E* ^+097^	.923	10.458
Focal abnormality	16949.11	0.00–6.6*E* ^+096^	.929	9.738

Diagnosis of dementia: 0 = VaD; 1 = AD.

**Table 3:** Predictive factors in Cox Proportionate Hazards Models, including diagnosis of dementia, age, educational level, sex, family history of dementia, CT scan findings, and vascular risk factors, for the progression of dementia (worsening of severity according to CDR scale).

Variable	Hazards ratio	CI 95% lower–upper	*P*-value	*r*
Diagnosis of dementia	18.627	0.94–370.77	.055	2.925
Age	1.060	96–1.17	.254	0.058
Sex	0.510	0.13–1.96	.327	−0.673
Educational level	1.323	1.10–1.59	.002	0.280
Vascular risk factors			.329	
Hypertension	38.32	1.37–1070.3	.032	3.646
Diabetes mellitus	0.000	0.00–2.3*E* ^+101^	.992	−9.928
Hypertension + diabetes mellitus	28.30	0.74 – 1082.7	.072	3.343
Family history of dementia			.339	
Present	0.473	0.09–2.62	.392	−0.748
Unknown	0.305	0.05–1.81	.191	−1.187
CT findings			.985	
Mild diffuse atrophy AND/OR ↑ ventricles	547201.7	0.00–8.5*E* ^+111^	.919	13.213
Atrophy + leukoaraiosis	728853.1	0.00–1.1*E* ^+117^	.918	13.499
Focal abnormality	392780.9	0.00 –6.1*E* ^+116^	.921	12.881

Diagnosis of dementia: 0 = VaD; 1= AD.
